# Efficacy and safety of fixed doses of intranasal Esketamine as an add-on therapy to Oral antidepressants in Japanese patients with treatment-resistant depression: a phase 2b randomized clinical study

**DOI:** 10.1186/s12888-021-03538-y

**Published:** 2021-10-25

**Authors:** Nagahide Takahashi, Aya Yamada, Ayako Shiraishi, Hiroko Shimizu, Ryosuke Goto, Yushin Tominaga

**Affiliations:** 1 Department of Psychiatry, Nagoya University Graduate School of Medicine, Nagoya, Japan; 2 Janssen Pharmaceutical K.K, 5-2, Nishikanda 3-chome, Chiyoda-ku, Tokyo, 101-0065 Japan

**Keywords:** N-methyl-D-aspartate receptor antagonist, Esketamine, Treatment-resistant depression, Nasal spray, Add-on therapy, Efficacy, Safety

## Abstract

**Background:**

Esketamine nasal spray (Spravato) in conjunction with oral antidepressants (ADs) is approved in the European Union, United States, and other markets for treatment-resistant depression (TRD). Efficacy, safety, and tolerability of esketamine nasal spray in Japanese patients with TRD needs to be assessed.

**Methods:**

This Phase 2b, randomized, double-blind (DB), placebo-controlled study was conducted in adult Japanese patients with TRD meeting the Diagnostic and Statistical Manual of Mental Disorders (fifth edition) criteria of major depressive disorder with nonresponse to ≥ 1 but < 5 different ADs in the current episode at screening. Patients were treated with a new oral AD for 6 weeks (prospective lead-in phase); nonresponders were randomized (2:1:1:1) to placebo or esketamine (28-, 56-, or 84-mg) nasal spray along with the continued use of AD for 4 weeks (DB induction phase). Responders (≥50% reduction from baseline in the Montgomery-Asberg Depression Rating Scale [MADRS] total score) from the DB induction phase continued into the 24-week posttreatment phase and patients who relapsed could participate in a 4-week open-label (OL) second induction (flexibly-dosed esketamine). The primary efficacy endpoint, change from baseline in the MADRS total score at Day 28 in the DB induction phase, was based on mixed-effects model using repeated measures pairwise comparisons using a Dunnett adjustment.

**Results:**

Of the 202 patients randomized in the DB induction phase (esketamine [*n* = 122] or placebo [*n* = 80]), the MADRS total scores decreased from baseline to Day 28 of the DB induction phase (− 15.2, − 14.5, − 15.1, and − 15.3 for esketamine 28 mg, 56 mg, 84 mg, and placebo groups, respectively), indicating an improvement in depressive symptoms; however, the difference between the esketamine and placebo groups was not statistically significant. The most common treatment-emergent adverse events during the DB induction phase in the combined esketamine group (incidences ranging from 12.3 to 41.0%) were blood pressure increased, dissociation, dizziness, somnolence, nausea, hypoaesthesia, vertigo, and headache; the incidence of each of these events was > 2-fold higher than the corresponding incidence in the placebo group.

**Conclusions:**

Efficacy of esketamine plus oral AD in Japanese TRD patients was not established; further investigation is warranted. All esketamine doses were safe and tolerated.

**Trial registration:**

ClinicalTrials.gov Identifier: NCT02918318. Registered: 28 September 2016.

## Background

Major depressive disorder (MDD) is a recurrent and disabling psychiatric illness associated with mortality and total years lost due to disability [1–3]. Depression is reported in > 264 million people globally, affecting women more than men [4]. The 12-month prevalence of MDD in Japan is 2.2% and the lifetime prevalence is 6.1 to 6.6%. The prevalence in Japan is lower than the United States (US)/European Union (EU) but the degree of burden on these populations is similar [5–7], and Japan is reported to be one of the countries with highest suicide rates (14.3 per 100,000 persons) according to the World Health Organization [8].

Despite treatment with multiple antidepressants (ADs), ⁓10 to 30% of patients do not achieve remission and develop treatment-resistant depression (TRD) [9]. TRD is defined as no response to at least 2 different ADs taken at adequate dosage and for adequate duration [10]. In all controlled Phase 3 studies, treatment resistance was defined in accordance with the regulatory definition ie, a lack of clinically meaningful improvement (defined for Phase 3 studies as ≤ 25%) in the current episode of depression after treatment with at least 2 different ADs prescribed in adequate dosages for an adequate duration (defined for Phase 3 studies as at least 6 weeks) [11, 12]. The data for incidence or prevalence of TRD in Japanese population are limited but a retrospective study of claims database estimated that 12% of pharmaceutically-treated depression patients develop TRD within a year [13]. Therefore, there is a significant need to develop novel treatment options that provide relief of depressive symptoms in Japanese patients with TRD.

The antidepressant effects of ketamine, a glutamate N-methyl-D-aspartate (NMDA) antagonist, are well-documented [14]. Esketamine, the S-enantiomer of racemic ketamine, has a greater affinity for the NMDA receptor than the R-enantiomer [15]. Esketamine nasal spray (Spravato) is currently approved in the EU, the US, and other markets for the treatment of adults with TRD when used in conjunction with traditional oral ADs [16–19]. Data from global studies of esketamine nasal spray have demonstrated rapid onset and maintenance of antidepressant effects in patients with TRD and in those with MDD who are at imminent risk for suicide [12, 20–25].

In a prior early study of esketamine nasal spray that included Japanese population, signals suggesting potential for efficacy were observed [26]. The current study was designed to further explore the efficacy and safety of esketamine nasal spray in Japanese patients, and to investigate the appropriate doses for the population.

## Methods

### Study design

**Fig. 1:**
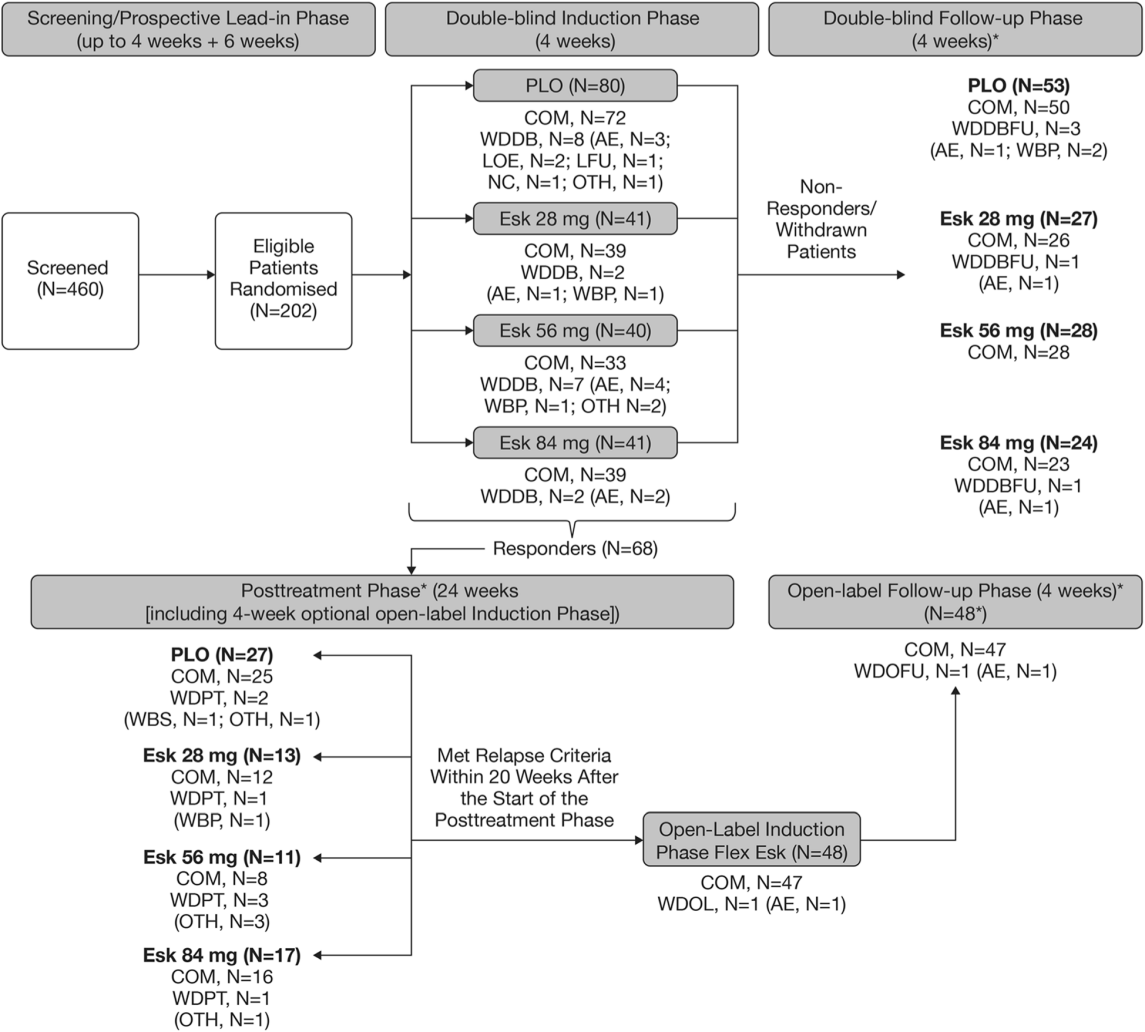
Patient Disposition. Notes: In the DB follow-up phase and posttreatment phase, the dose groups are the ones randomized during the DB induction phase. *The patients received only oral AD medication during the DB follow-up phase, posttreatment phase, and OL follow-up phase. Abbreviations: AD = antidepressant; AE = adverse event; COM = completed; DB = double-blind; Esk = esketamine; Flex = flexible; LFU = lost to follow-up; LOE = loss of efficacy; N = number of patients; NC = noncompliance; OL = open-label; OTH = other reason for withdrawal; PLO = placebo; WBP = withdrawal by patient; WDDB = withdrawal from DB induction phase; WDDBFU = withdrawal from DB follow-up phase; WDOL = withdrawal from OL induction phase; WDOLFU = withdrawal from OL follow-up phase; WDPT = withdrawal from posttreatment phase

This was a Phase 2b, randomized, double-blind (DB), placebo-controlled, multicenter study consisting of the following phases: 1) a 4-week screening phase; 2) a 6-week open-label (OL) prospective lead-in phase; 3) a 4-week DB induction phase; 4) up to 24-week posttreatment phase including an optional 4-week OL induction phase; 5) and a 4-week follow-up phase (Fig. 1).

During the prospective lead-in phase, patients received a new OL oral AD therapy; nonresponders to this oral AD (defined as those who achieved ≤ 25% improvement in the Montgomery-Asberg Depression Rating Scale [MADRS] total score during the prospective lead-in phase and at randomization) and patients with a MADRS total score of ≥ 28 at 2 weeks before randomization and at randomization were considered to meet the definition of TRD, and entered the 4-week DB induction phase.

In the DB induction phase, patients were randomized to either placebo or fixed-dose esketamine (28-, 56-, or 84-mg) groups (2:1:1:1; hereafter referred to as Esk28, Esk56, and Esk84, respectively), and received placebo or esketamine nasal spray on top of the oral AD that was continued unchanged from the prospective lead-in phase.

At the end of the DB induction phase, responders (defined as those who achieved ≥ 50% reduction from baseline in the MADRS total score) were eligible for the 24-week posttreatment phase, and nonresponders/withdrawn patients entered the follow-up phase. Responders from the DB induction phase who relapsed (i.e. had MADRS total scores ≥ 22 for 2 consecutive assessments, or hospitalization for worsening depression, or any other clinically relevant event determined per clinical judgment to be suggestive of a relapse) within 20 weeks after the start of the posttreatment phase were eligible to enter the OL induction phase and receive flexible OL esketamine doses (Esk28, Esk56, or Esk84), starting from Esk56. All patients who were randomized in the DB induction phase had a follow-up of 4 weeks duration, unless they withdrew consent. During the follow-up phase, where standard of care was allowed, oral AD was continued, unless determined as not clinically appropriate.

### Patient population

Japanese patients (aged 20 to 64 years) who had a single episode (≥ 2 years) or recurrent MDD (as per Diagnostic and Statistical Manual of Mental Disorders [fifth edition] criteria, DSM-5) [27], without psychotic features, confirmed by the Mini International Neuropsychiatric Interview, and were nonresponders (≤ 25% improvement in the MADRS total score) to ≥ 1 but < 5 different oral ADs (based on Massachusetts General Hospital-Antidepressant Treatment Response Questionnaire [MGH-ATRQ]) in the current episode were enrolled. Patients with current or prior DSM-5 diagnosis of a psychotic disorder or MDD with psychotic features, bipolar or related disorders, or patients who had homicidal ideation/intent, or suicidal ideation with some intent to act within 6 months of screening or those with history of suicidal behavior within the past year were excluded.

### Randomization procedure

Patients were centrally randomized based on a computer-generated randomization schedule. The randomization was balanced by using randomly permuted blocks across the 4 treatment groups.

### Newly initiated Oral antidepressant

A new, OL oral AD medication (escitalopram, paroxetine controlled-release, sertraline, duloxetine, venlafaxine extended-release, or mirtazapine) was initiated for all patients in the prospective lead-in phase based on the clinical guideline of MDD published by Japanese Society of Mood Disorders [28]. The investigator assigned the AD medication based on the review of the MGH-ATRQ and relevant prior AD medication. The ADs to which a patient had not previously responded (in the current depressive episode), or did not tolerate (lifetime) were not used.

### Intranasal study drug administration

During the DB induction phase, dosing occurred twice weekly for 4 weeks. Patients assigned to Esk84 started at Esk56 on Day 1 with fixed up-titration to Esk84 on Day 4. No dose adjustments were permitted thereafter.

During the OL induction phase, patients received Esk56 on Day 1 with an up-titration to Esk84 on Day 4. Based on efficacy and tolerability, the dose could be reduced or increased by 28 mg on Days 8 and 11 or maintained. On Day 15, only dose reduction was permitted for tolerability; no dose increase was permitted. After Day 15, the dose was to remain stable; however, if needed for tolerability, a dose reduction was permitted until Day 25.

### Efficacy evaluations

The primary efficacy endpoint was change from baseline in the MADRS total score (7-day recall) to the end of 4-week DB induction phase, performed by independent central remote rater through telephone interview to assess the severity of depression using the Structured Interview Guide for the MADRS (SIGMA: Williams 2008) [29].

Secondary efficacy evaluations included proportion of responders (≥ 50% reduction from baseline in MADRS total score) and remitters (MADRS total score ≤ 12); changes from baseline in the Clinical Global Impression-Severity Scale (CGI-S, clinician-rated scale) [30] and Sheehan Disability Scale (SDS, patient-reported scale) total score; all in the DB induction phase [31, 32]; and time to relapse (time between the end of the DB induction phase and the first documentation of a relapse event during the posttreatment phase).

### Safety evaluations

Safety evaluations included monitoring of adverse events (AEs), vital signs measurements, clinical laboratory tests, electrocardiogram (ECG), Clinician Administered Dissociative States Scale (CADSS) to assess treatment-emergent dissociative symptoms [33], Modified Observer’s Assessment of Alertness/Sedation (MOAA/S) to measure treatment-emergent sedation [34], Columbia-Suicide Severity Rating Scale (C-SSRS) to assess suicidal ideation and behavior [35], Brief Psychiatric Rating Scale (BPRS+) to assess treatment-emergent psychotic symptoms [36], and Physician Withdrawal Checklist 20-item (PWC-20) to assess potential withdrawal symptoms following cessation of study drug [37].

### Statistical methods

#### Sample size determination

A sample size of 183 patients (72 in the placebo and 37 in each esketamine group) was required to achieve 80% power to detect difference for at least 1 dose group of esketamine to placebo using a Dunnett adjustment (assuming MADRS total score difference for the DB induction phase of 4 to 5 points between each dose of esketamine and the placebo, a standard deviation [SD] of 10 for each treatment group, a 1-sided significance level of 0.05, and a drop-out rate of 12.5%). The treatment difference and SD used in this calculation were assumed based on results of the Japanese panel (Esk 14 mg and Esk56) of the NCT01998958 study [26] with clinical consideration.

#### Efficacy analyses

Full analysis sets (FAS): Efficacy summaries for the DB and OL induction phases were based on the FAS (DB) and FAS (OL) analysis sets, defined as all randomized patients who received at least 1 dose of study drug during the DB and OL induction phases, respectively. The FAS (responders), defined as all randomized patients who received at least 1 dose of study drug during the DB induction phase and who were responders at the end of the DB induction phase and entered the posttreatment phase, was used for the summaries in the posttreatment phase and for the analysis of time to relapse.

Follow-up analysis sets: Efficacy summaries for the DB and OL follow-up phases were based on follow-up (DB) and follow-up (OL) analysis sets, defined as all patients who did not respond at the end of DB induction phase and entered the DB follow-up phase and patients who entered the OL follow-up phase, respectively.

The primary efficacy analysis was based on the FAS (DB) analysis set. The primary efficacy endpoint, change from baseline in the MADRS total score at Day 28 in the DB induction phase was based on the mixed-effects model using repeated measures (MMRM) pairwise comparisons (each esketamine group with the placebo group) using a Dunnett adjustment. To analyze the dose-response relationship, a multiple comparison procedure-modeling (MCP-Mod) approach was applied to the MMRM estimates. A multiple trend test was performed using 5 models (E_max_ [the maximum effect attributable to the drug], sigmoid E_max_, linear, exponential, and quadratic) with an overall significance level of 5% (1-sided) [38].

For all other analyses of the primary and secondary efficacy endpoints, no multiplicity adjustment was done.

The proportion of responders/remitters was summarized at each time point during the DB induction phase. Descriptive statistics was provided for the CGI-S and SDS. Time to relapse was estimated by the Kaplan-Meier method. No comparison between esketamine and placebo was made because the objective of this posttreatment period was to explore the durability of response after 4-week esketamine treatment.

#### Safety analyses

The safety analysis sets included all randomized patients who received at least 1 dose of study drug in the DB (safety [DB]) or OL (safety [OL]) induction phases. Safety summaries for the follow-up phases were based on the follow-up analysis sets. Descriptive statistics were provided for all safety evaluations. Adverse events were coded using the Medical Dictionary for Regulatory Activities (MedDRA), version 22.0.

## Results

### Study population

The study was conducted from 14 December 2016 to 13 December 2019 at 58 sites in Japan. Of the 460 patients screened, 308 (67.0%) entered the prospective lead-in phase, 202 (43.9%) were randomized in the DB induction phase (placebo [*n* = 80], Esk28 [*n* = 41], Esk56 [*n* = 40], and Esk84 [*n* = 41]), and 183/202 (90.6%) completed the DB induction phase (Fig. 1). A total of 19/202 (9.4%) patients were withdrawn from the DB induction phase: 2/41 (4.9%), 7/40 (17.5%), 2/41 (4.9%), and 8/80 (10.0%) from the Esk28, Esk56, Esk84, and placebo groups, respectively, with the most common reason being AEs across all treatment groups (10/202 [5.0%] patients).

The demographic and baseline characteristics were generally comparable across the treatment groups. The proportion of males and females was 52.5 and 47.5%, respectively, and the mean (SD) age was 43.4 (10.35) years (Table 1).

**Table 1:** Demographics and Baseline Characteristics

Category	Esk28 (***N =*** 41)	Esk56 (***N =*** 40)	Esk84 (***N =*** 41)	Placebo (***N =*** 80)	Total (***N*** = 202)
Age (years)					
Mean (SD)	45.9 (9.97)	42.5 (8.36)	41.9 (10.26)	43.3 (11.40)	43.4 (10.35)
Sex, n (%)					
Male	18 (43.9%)	24 (60.0%)	23 (56.1%)	41 (51.3%)	106 (52.5%)
Female	23 (56.1%)	16 (40.0%)	18 (43.9%)	39 (48.8%)	96 (47.5%)
Baseline BMI (kg/m^2^)					
Mean (SD)	25.81 (4.733)	24.37 (5.388)	23.86 (3.949)	25.01 (4.729)	24.81 (4.735)
Hypertension status^a^, n (%)					
Yes	5 (12.2%)	11 (27.5%)	9 (22.0%)	10 (12.5%)	35 (17.3%)
No	36 (87.8%)	29 (72.5%)	32 (78.0%)	70 (87.5%)	167 (82.7%)
Oral antidepressant, n (%)					
Duloxetine	1 (2.4%)	4 (10.0%)	4 (9.8%)	11 (13.8%)	20 (9.9%)
Venlafaxine XR	9 (22.0%)	7 (17.5%)	4 (9.8%)	10 (12.5%)	30 (14.9%)
Escitalopram	18 (43.9%)	11 (27.5%)	17 (41.5%)	25 (31.3%)	71 (35.1%)
Sertraline	8 (19.5%)	11 (27.5%)	10 (24.4%)	23 (28.8%)	52 (25.7%)
Paroxetine CR	2 (4.9%)	6 (15.0%)	1 (2.4%)	5 (6.3%)	14 (6.9%)
Mirtazapine	3 (7.3%)	1 (2.5%)	5 (12.2%)	6 (7.5%)	15 (7.4%)
Age when diagnosed with MDD (years)					
Mean (SD)	37.9 (10.80)	33.5 (9.51)	32.8 (9.76)	34.4 (11.18)	34.6 (10.58)

^a^Hypertension status = Yes, if SBP ≥ 140 mmHg and/or DBP ≥ 90 mmHg at least one time point before DB Induction Phase. Hypertension status = No, if SBP < 140 mmHg and DBP < 90 mmHg at all time points before the DB Induction Phase

*Abbreviations*: *BMI* body mass index, *CR* controlled-release, *DB* double-blind, *DBP* diastolic blood pressure, *Esk* esketamine, *MDD* major depressive disorder, *N* number of patients, *n* subset of patients, *SBP* systolic blood pressure, *SD* standard deviation, *XR* extended-release

At baseline, the mean (SD) MADRS total score was 37.5 (5.64), the majority of patients were moderately ill (45.5% [92/202]) based on the CGI-S scores, and 19.8% (40/202) of patients had suicidal ideation based on the C-SSRS. The mean (SD) duration of the current episode of depression was 63.5 (103.04) weeks. At baseline, 1.5% (3/202), 65.8% (133/202), and 32.7% (66/202) of patients had 1, 2 to 3, and > 3 depressive episodes, including the current episode, respectively. Prior to randomization, 51.5% (104/202) and 48.5% (98/202) of patients had 2 and ≥ 3 previous treatments in the current episode, respectively, including those who switched at the beginning of the prospective lead-in phase (Table 2).

**Table 2:** Baseline Psychiatric History

Category	Esk28 (***N*** = 41)	Esk56 (***N*** = 40)	Esk84 (***N*** = 41)	Placebo (***N*** = 80)	Total (***N*** = 202)
Baseline MADRS total score					
Mean (SD)	38.4 (6.07)	37.9 (5.41)	35.9 (5.28)	37.7 (5.65)	37.5 (5.64)
Baseline CGI-S score					
Mean (SD)	4.7 (0.69)	4.7 (0.73)	4.7 (0.75)	4.7 (0.79)	4.7 (0.75)
Baseline CGI-S^a^, n (%)					
Moderately ill	18 (43.9%)	19 (47.5%)	19 (46.3%)	36 (45.0%)	92 (45.5%)
Markedly ill	18 (43.9%)	15 (37.5%)	15 (36.6%)	31 (38.8%)	79 (39.1%)
Severely ill	5 (12.2%)	6 (15.0%)	7 (17.1%)	11 (13.8%)	29 (14.4%)
extremely ill patients	0	0	0	2 (2.5%)	2 (1.0%)
Baseline C-SSRS^b^, n (%)					
No event	33 (80.5%)	33 (82.5%)	36 (87.8%)	60 (75.0%)	162 (80.2%)
Suicidal ideation	8 (19.5%)	7 (17.5%)	5 (12.2%)	20 (25.0%)	40 (19.8%)
Duration of current episode at screening (weeks)					
Mean (SD)	73.9 (115.38)	55.5 (46.99)	65.0 (98.93)	61.4 (118.89)	63.5 (103.04)
Number of previous treatments in current episode^c^, n (%)					
2	19 (46.3%)	22 (55.0%)	21 (51.2%)	42 (52.5%)	104 (51.5%)
3 or more	22 (53.7%)	18 (45.0%)	20 (48.8%)	38 (47.5%)	98 (48.5%)
Number of episodes including current episode, n (%)					
1	1 (2.4%)	0	1 (2.4%)	1 (1.3%)	3 (1.5%)
2–3	25 (61.0%)	30 (75.0%)	22 (53.7%)	56 (70.0%)	133 (65.8%)
> 3	15 (36.6%)	10 (25.0%)	18 (43.9%)	23 (28.8%)	66 (32.7%)
Had been considered to be eligible for electroconvulsive therapy, n (%)					
Yes	10 (24.4%)	9 (22.5%)	11 (26.8%)	15 (18.8%)	45 (22.3%)
No	31 (75.6%)	28 (70.0%)	25 (61.0%)	62 (77.5%)	146 (72.3%)
Unknown	0	3 (7.5%)	5 (12.2%)	3 (3.8%)	11 (5.4%)

^a^The CGI-S evaluates the severity of psychopathology on a scale of 0 to 7. Considering total clinical experience, a patient was assessed on severity of mental illness at the time of rating according to: 0 = not assessed; 1 = normal (not at all ill); 2 = borderline mentally ill; 3 = mildly ill; 4 = moderately ill; 5 = markedly ill; 6 = severely ill; and 7 = among the most extremely ill patients

^b^C-SSRS category: No event = 0; Suicidal ideation = 1, 2, 3, 4, 5; Suicidal behavior = 6, 7, 8, 9, 10

^c^Number of AD medications taken for at least 6 weeks during the current episode as obtained from MGH-ATRQ and plus 1 for the new oral AD taken at screening phase

*Abbreviations*: *AD* antidepressant, *CGI-S* Clinical Global Impression-Severity, *C-SSRS* Columbia-Suicide Severity Rating Scale, *Esk* esketamine, *MADRS* Montgomery-Asberg Depression Rating Scale, *MGH-ATRQ* Massachusetts General Hospital–Antidepressant Treatment Response Questionnaire, *N* number of patients, *SD* standard deviation

### Efficacy findings

#### Primary efficacy analyses

**Fig. 2:**
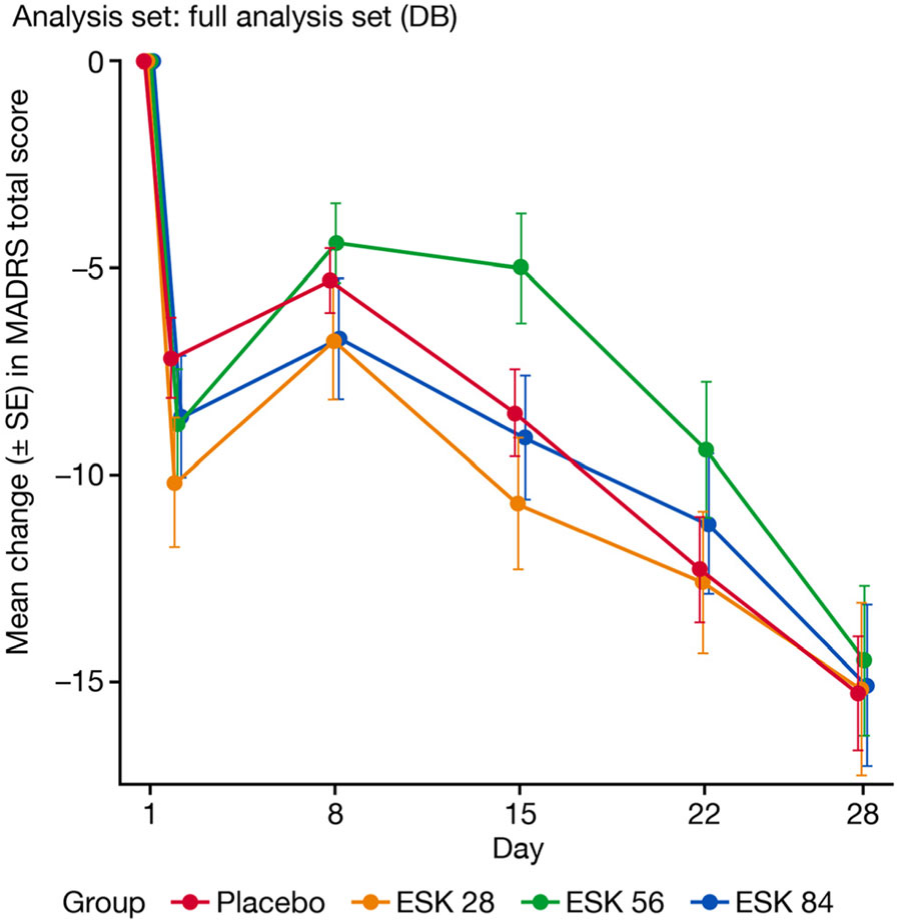
Mean Change in MADRS Total Score Over Time Observed Case MMRM; DB Induction Phase. Abbreviations: DB = double-blind; Esk = esketamine; MADRS = Montgomery-Asberg Depression Rating Scale; MMRM = mixed-model for repeated measures; SE = standard error

The mean MADRS total score decreased (indicating improvement) from baseline to Day 28 (− 15.2, − 14.5, − 15.1, and − 15.3 for Esk28, Esk56, Esk84 and placebo groups, respectively), with comparable improvement across all treatment groups (Table 3; Fig. 2). Based on the MMRM model, the least-squares (LS) mean differences (standard error [SE]) of the changes in the MADRS total score between the Esk28, Esk56, and Esk84, and placebo groups were - 1.0 (2.25), 0.6 (2.33), and - 0.9 (2.26), respectively. The improvement in the esketamine groups compared with the placebo group did not reach statistical significance (1-sided *p* = 0.475, *p* = 0.504, and *p* = 0.482, respectively) (Table 3).

**Table 3:** MADRS Total Score: Change From Baseline to Day 28 MMRM; DB Induction Phase

Category	Esk28 (***N =*** 41)	Esk56 (***N =*** 40)	Esk84 (***N =*** 41)	Placebo (***N =*** 80)
Baseline				
N	41	40	41	80
Mean (SD)	38.4 (6.07)	37.9 (5.41)	35.9 (5.28)	37.7 (5.65)
Median (Range)	36.0 (28; 58)	37.5 (29; 49)	35.0 (28; 47)	37.0 (29; 51)
Day 28				
N	39	34	39	72
Mean (SD)	22.9 (12.46)	23.6 (11.01)	21.0 (11.24)	22.4 (11.43)
Median (Range)	24.0 (0; 55)	22.0 (2; 47)	19.0 (1; 43)	21.5 (1; 46)
Change from baseline to Day 28 (DB)				
N	39	34	39	72
Mean (SD)	- 15.2 (13.07)	- 14.5 (10.53)	- 15.1 (12.21)	- 15.3 (11.68)
Median (Range)	- 13.0 (− 52; 7)	- 15.0 (− 40; 5)	- 15.0 (− 44; 5)	- 13.0 (− 50; 4)
MMRM analysis^a^				
Diff. of LS means (SE) (Esk minus Placebo)	- 1.0 (2.25)	0.6 (2.33)	- 0.9 (2.26)	
90% confidence interval on diff^b^	- 5.77; 3.70	- 4.32; 5.47	- 5.66; 3.83	
1-sided *p-*value (Esk minus Placebo)^b^	0.475	0.504	0.482	

MADRS total score ranges from 0 to 60; a higher score indicates a more severe condition

Negative change in score indicates improvement.

Results based on sensitivity analyses (ANCOVA LOCF model and MMRM analyses based on follow-up data were consistent with the primary MMRM analysis).

^a^Test for no difference between treatments based on MMRM with change from baseline as the response variable and the fixed effect model terms for treatment (Esk28, Esk56, Esk84, Placebo), day and treatment-by-day, and baseline value as a covariate. A negative difference favors esketamine

^b^Confidence interval and *p*-value are based on the Dunnett adjustment

*Abbreviations*: *ANCOVA* analysis of covariance, *DB* double-blind, *diff* difference, *Esk* esketamine, *LOCF* last observation carried forward, *LS* least-square, *MADRS* Montgomery-Asberg depression rating scale, *MMRM* mixed-model for repeated measures, *N* number of patients, *SD* standard deviation, *SE* standard error

The MCP-Mod analysis models did not show a significant dose-response relationship in the change from baseline in the MADRS total score at Day 28 in all 5 prespecified models.

#### Secondary efficacy analyses

On Day 28 of the DB induction phase, the proportion of responders based on the MADRS total score was 33.3% (13/39 patients), 35.3% (12/34 patients), 43.6% (17/39 patients), and 37.5% (27/72 patients) in the Esk28, Esk56, Esk84, and placebo groups, respectively. The proportion of remitters was 23.1% (9/39 patients), 11.8% (4/34 patients), 23.1% (9/39 patients), and 20.8% (15/72 patients) in the Esk28, Esk56, Esk84, and placebo groups, respectively.

**Fig. 3:**
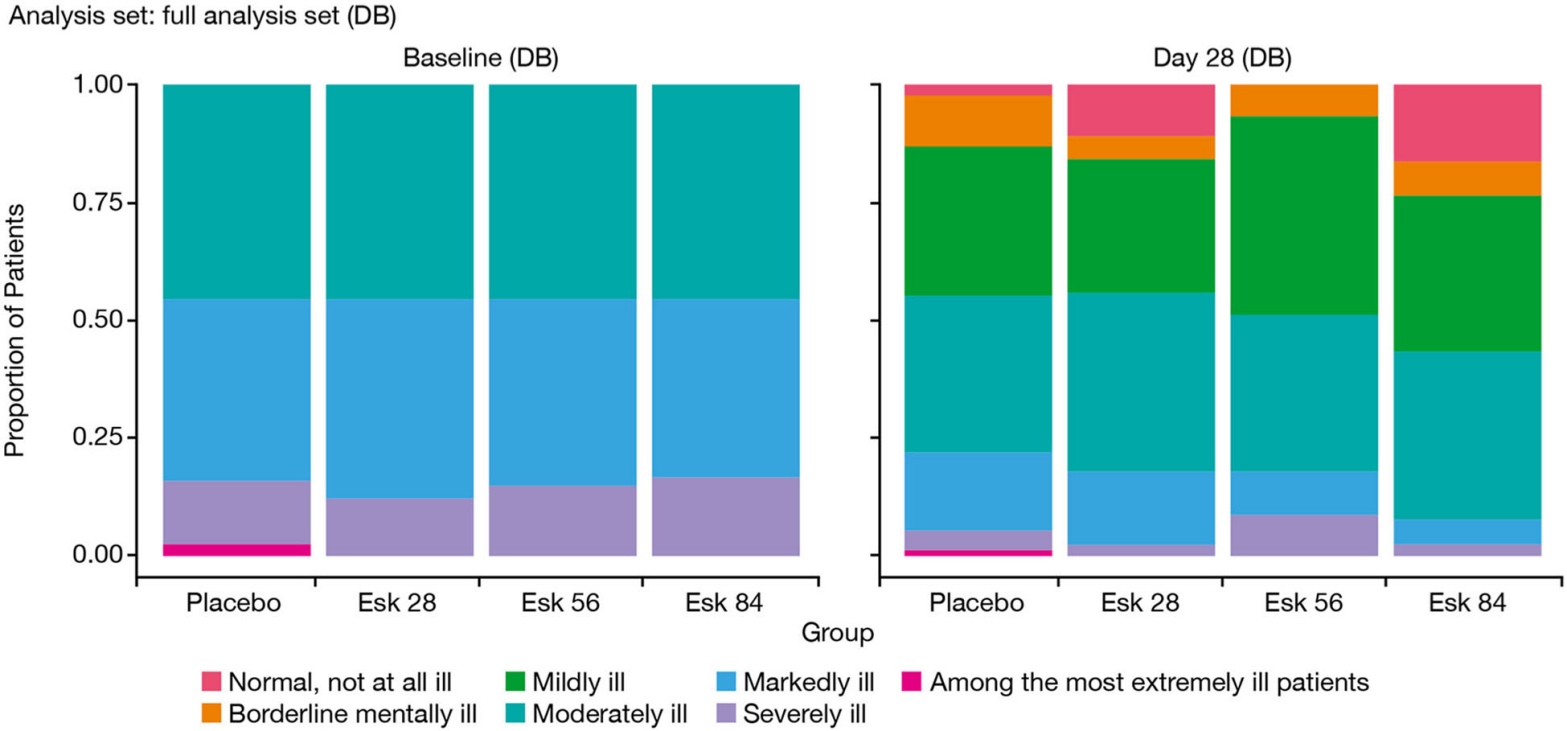
CGI-S Score Over Time; DB Induction Phase. Abbreviations: CGI-S=Clinical Global Impression-Severity; DB = double-blind; Esk = esketamine

The severity of illness (CGI-S) improved over time in all esketamine and placebo groups from baseline to Day 28 of the DB induction phase (Fig. 3). Functional impairment and associated disability (SDS total scores) improved over time in all esketamine and placebo groups from baseline to Day 28 of the DB induction phase. The mean (SD) decrease in the SDS total score was 8.6 (8.68), 7.9 (7.94), 9.5 (8.93), and 7.0 (7.39) in the Esk28, Esk56, Esk84, and placebo groups, respectively.

During the posttreatment phase, the median time to relapse for all remitters (MADRS total score ≤ 12) and responders (≥ 50% reduction from baseline in the MADRS total score) but who were not in remission was 34.0 days (90% confidence interval [CI]: 26.0; 71.0) and 44.0 days (90% CI: 22.0; 100.0) in the combined esketamine group, respectively.

During the OL induction phase, the mean MADRS total score decreased from baseline to Day 28 (decrease of 14.3 and 15.6, respectively, in patients receiving esketamine and placebo in the DB induction phase). Overall, a decrease was seen in the MADRS total score from baseline (OL) to the OL induction phase endpoint; no evidence of tolerance was seen after the second induction treatment in patients who received any active esketamine in DB induction phase. On Day 28 of the OL induction phase, the proportion of responders and remitters (based on the MADRS total score) in the flexible esketamine group was 44.7% (21/47 patients) and 42.6% (20/47 patients), respectively.

### Safety findings

Overall, the incidence of treatment-emergent adverse events (TEAEs) was higher in all esketamine groups compared with the placebo group during the DB induction phase (Table 4). The most common TEAEs (≥ 10% of patients) reported during the DB induction phase in the combined esketamine group were blood pressure (BP) increased, dissociation, dizziness, somnolence, nausea, hypoaesthesia, vertigo, and headache (incidence range: 12.3 to 41.0%). The incidence of each TEAE was 2 to 12 times higher in combined esketamine group as compared to the placebo group. Most of these TEAEs were mild (65.6% for combined esketamine and 52.5% for placebo groups) or moderate (19.7% for combined esketamine and 10.0% for placebo groups) in severity. Dissociation and sedation (both resolved on the day of onset) were the only severe TEAEs reported in 2 or more patients in the combined esketamine group (higher in Esk84 group), and suicidal ideation (1 patient; duration 57 days) was the only severe TEAE reported in the placebo group. The TEAE incidence profile during the OL induction phase was similar to the DB induction phase.

**Table 4:** TEAEs in At least 5% of Patients in any Treatment Group; DB Induction Phase

	Esk28 (***N =*** 41)	Esk56 (***N*** = 41)^**a**^	Esk84 (***N*** = 40)	Comb Esk (***N*** = 122)	Placebo (***N*** = 80)
**Patients with TEAEs, n (%)**	**33 (80.5%)**	**39 (95.1%)**	**39 (97.5%)**	**111 (91.0%)**	**51 (63.8%)**
Blood pressure increased	12 (29.3%)	19 (46.3%)	19 (47.5%)	50 (41.0%)	8 (10.0%)
Dissociation	14 (34.1%)	10 (24.4%)	22 (55.0%)	46 (37.7%)	7 (8.8%)
Dizziness	11 (26.8%)	18 (43.9%)	15 (37.5%)	44 (36.1%)	5 (6.3%)
Somnolence	10 (24.4%)	13 (31.7%)	11 (27.5%)	34 (27.9%)	14 (17.5%)
Nausea	7 (17.1%)	7 (17.1%)	8 (20.0%)	22 (18.0%)	7 (8.8%)
Hypoaesthesia	7 (17.1%)	8 (19.5%)	5 (12.5%)	20 (16.4%)	4 (5.0%)
Vertigo	4 (9.8%)	7 (17.1%)	8 (20.0%)	19 (15.6%)	1 (1.3%)
Headache	6 (14.6%)	5 (12.2%)	4 (10.0%)	15 (12.3%)	3 (3.8%)
Asthenia	2 (4.9%)	7 (17.1%)	3 (7.5%)	12 (9.8%)	0
Sedation	1 (2.4%)	4 (9.8%)	5 (12.5%)	10 (8.2%)	0
Vomiting	1 (2.4%)	3 (7.3%)	4 (10.0%)	8 (6.6%)	3 (3.8%)
Feeling drunk	1 (2.4%)	5 (12.2%)	2 (5.0%)	8 (6.6%)	1 (1.3%)
Euphoric mood	0	4 (9.8%)	3 (7.5%)	7 (5.7%)	0
Hypoaesthesia oral	3 (7.3%)	2 (4.9%)	2 (5.0%)	7 (5.7%)	0
Diarrhoea	0	4 (9.8%)	2 (5.0%)	6 (4.9%)	3 (3.8%)
Malaise	0	3 (7.3%)	3 (7.5%)	6 (4.9%)	0
Dizziness postural	0	3 (7.3%)	2 (5.0%)	5 (4.1%)	0
Mental impairment	3 (7.3%)	2 (4.9%)	0	5 (4.1%)	0
Palpitations	1 (2.4%)	2 (4.9%)	2 (5.0%)	5 (4.1%)	1 (1.3%)
Diplopia	1 (2.4%)	3 (7.3%)	0	4 (3.3%)	0
Muscular weakness	0	2 (4.9%)	2 (5.0%)	4 (3.3%)	0
Dysarthria	1 (2.4%)	0	2 (5.0%)	3 (2.5%)	0
Hypotonia	0	0	3 (7.5%)	3 (2.5%)	0
Hallucination	0	1 (2.4%)	2 (5.0%)	3 (2.5%)	0
Suicidal ideation	0	1 (2.4%)	2 (5.0%)	3 (2.5%)	2 (2.5%)
Hyperacusis	0	1 (2.4%)	2 (5.0%)	3 (2.5%)	0
Oropharyngeal pain	0	1 (2.4%)	2 (5.0%)	3 (2.5%)	2 (2.5%)
Tinnitus	1 (2.4%)	0	2 (5.0%)	3 (2.5%)	0
Blood pressure diastolic increased	0	0	2 (5.0%)	2 (1.6%)	2 (2.5%)
Dyslalia	0	0	2 (5.0%)	2 (1.6%)	0
Respiratory rate decreased	0	0	2 (5.0%)	2 (1.6%)	0
Thirst	0	0	2 (5.0%)	2 (1.6%)	0
Weight increased	0	0	2 (5.0%)	2 (1.6%)	0

Incidence is based on the number of patients experiencing at least 1 adverse event, not the number of events

Adverse events are coded using MedDRA version 22.0

^a^1 patient was randomized to the Esk84 mg group but was dosed with Esk56 mg on Days 1 and 4 and 28 mg on Day 8, then was withdrawn due to an adverse event on Day 11. This patient is summarized under the Esk84 mg group for the efficacy analyses, and under the Esk56 group for the safety analyses

*Abbreviations*: *Comb* combined, *DB* double-blind, *Esk* esketamine, *MedDRA* Medical Dictionary for Regulatory Activities, *TEAE* treatment-emergent adverse event

In the DB induction phase, a transient dose-related elevation of CADSS score immediately after esketamine administration was reported with a peak at 40 min that spontaneously returned to predose values by1.5 h postdose. The highest (mean [SD]) CADSS total score was 5.5 (7.98), reported in the Esk84 group on Day 1. The peak value and the time course change of CADSS was similar in the OL induction phase. During the DB induction phase at any time, a higher proportion of patients in the Esk56 (12.2%) and Esk84 groups (17.5%) had MOAA/S score ≤ 3 (indicating moderate or greater sedation) compared with the Esk28 (4.9%) and placebo groups (0). A total of 8/48 (16.7%) patients had MOAA/S score ≤ 3 at any time during the OL induction phase.

There were no deaths after randomization. Serious TEAEs were reported in 3 patients in the DB induction phase (fracture [*n* = 1] with Esk28, and suicidal ideation with Esk84 and placebo [*n* = 1 each]). The events of fracture (Esk28 group) and suicidal ideation (Esk84 group) were assessed as not related and doubtfully related to the study drug, respectively. Ten patients were reported with TEAEs leading to discontinuation of study drug during the DB induction phase: *n* = 1 (2.4%) in the Esk28 group (fracture), *n* = 5 (12.2%) in the Esk56 group (*n =* 1 with dissociation, dizziness, and sedation; n = 1 with nausea, and sedation; and *n* = 1 each with depersonalization/derealization disorder, ventricular extrasystoles, and malaise), *n =* 1 (2.5%) in the Esk84 group (suicidal ideation), and *n* = 3 (3.8%) in the placebo group (*n =* 1 each, with suicidal ideation, amnesia, rhinorrhea). The serious TEAEs of fracture and suicidal ideation, and nonserious TEAE of malaise were reported as severe.

During the DB follow-up phase, serious AEs were reported in 3 patients: 2 patients previously treated with placebo (ankle fracture and suicide attempt, *n =* 1 each) and 1 patient previously treated with Esk84 (cerebral disorder [i.e. higher brain dysfunction] and muscular weakness). The AE of cerebral disorder (i.e. higher brain dysfunction) was reported 2 weeks after the patient’s last dose of esketamine (Day 38), and followed by a report of muscular weakness 2 months later (Day 100); both events were subsequently recategorized as serious AEs on Day 115. Although the causality of these events was assessed as probably related to esketamine by the investigator, based on the latent onset of events and negative neurological evaluations (including physical examination, magnetic resonance imaging [MRI], and electroencephalogram [EEG] findings), the sponsor assessed that these were unrelated to the study drug.

No clinically relevant changes were observed for laboratory and ECG results throughout the treatment and follow-up phases. The BP in all esketamine groups increased at 40 min postdose and returned close to the predose values by 1.5 h postdose during the DB induction phase. There was a greater increase in the Esk84 group than the other esketamine groups. During the DB induction phase, the average maximum increase in the mean systolic BP from predose to any postdose time point across all treatment days was 12.9, 17.2, 19.6, and 6.5 mmHg in Esk28, Esk56, Esk84, and placebo groups, respectively; the average maximum increase in the diastolic BP was 9.4, 12.0, 13.4, and 6.9 mmHg in Esk28, Esk56, Esk84, and placebo groups, respectively. Overall, the changes were similar in DB and OL induction phases.

Suicidal ideation and behavior improved in patients during the DB induction phase based on the C-SSRS assessment. At the DB endpoint, the proportion of patients reporting no suicidal ideation or behavior was higher or remained stable as compared to baseline in the Esk28 (95.1% vs 80.5%), Esk56 (95.0% vs 82.9%), Esk84 (87.5% vs 87.5%), and placebo (88.6% vs 75.0%) groups. No cases of treatment-emergent psychosis were observed in any patients with esketamine during the study based on the review of AEs and BPRS+. The changes in withdrawal symptoms assessed by the PWC-20 after cessation of treatment with esketamine were consistent with the observed changes in symptoms of depression and anxiety. No clear evidence of withdrawal symptoms was observed in either DB or OL induction phases after cessation of either esketamine or placebo based on the PWC-20 assessment. Of note, there were no reports of drug abuse or cravings during the follow-up phase.

## Discussion

This study evaluated fixed-dosed esketamine (28-, 56-, 84-mg) nasal spray as an adjunct to oral ADs in Japanese patients with TRD. An improvement in depressive symptoms across all esketamine and placebo groups was observed on Day 28; however, improvement in the esketamine groups compared with placebo did not reach a statistical significance (*p* > 0.05).

Failure to confirm the therapeutic effect in this study may be attributable to multiple factors that might have reduced the ability to detect efficacy signals, and these are discussed below:

### Differences in patients’ demographics and baseline disease characteristics

As compared to global Phase 3 studies in which the efficacy of esketamine was observed [12, 23], there were some differences in the clinical backgrounds of patients (e.g. this study included predominantly male patients, the patients had lower baseline severity of CGI-S, fewer number of previous treatments, and shorter duration of current episode). Nonetheless, the average baseline MADRS score after the prospective lead-in phase (37.5) was consistent with that observed in the non-Japanese population [12, 23] and reflective of severe depressive symptoms.

### Study design and patient expectation of treatment benefit

It has been reported that patient expectation of benefit from the treatment is associated with a placebo response [39]. Several design aspects of this study may have influenced the patients’ expectation.

First, placebo response has been reported to be negatively correlated with the probability of receiving placebo [39, 40].A randomization ratio of 60% (esketamine) to 40% (placebo) may have raised patients’ expectations of the likelihood of receiving the active treatment, leading to a high placebo response.

Other design elements that may have influenced patients’ expectation include criteria for study continuation into both DB induction phase and posttreatment phase at the end of DB induction phase as discussed below.

To be eligible for randomization into the DB induction phase, the prospective lead-in phase had rigid MADRS criteria (no response and MADRS total score of ≥ 28 at 2 visits prior to randomization and at randomization). In order to be eligible to receive the study drug, the baseline MADRS total scores among some patients may have been inflated, potentially contributing to a large decrease in the MADRS total score in the placebo group. This is supported by the observation that a subset of patients (32/202 [15.8%]) had baseline CGI-S scores that were lower than would be expected given their corresponding MADRS scores (data not shown). Inflation of baseline scores is known to distort the measurement of change from baseline, increase variability, and have a significant impact on efficacy signal detection [41].

Furthermore, at the end of the DB induction phase, only responders were eligible to participate in the OL induction phase. It is conceivable that making the possibility to continue treatment contingent on response might have also influenced ratings. This is supported by the observation that a few patients in both esketamine and placebo groups showed a sudden and steep improvement only for 1 or 2 visits at the end of the DB induction phase, then their symptoms rapidly returned to the baseline values after they proceeded to the posttreatment phase. This is an unlikely course of depression and contrary to the findings observed in the global short-term studies [12, 23].

Additionally, patient expectation could have been augmented by frequent site visits (twice a week in the present study) [42], esketamine having a new mechanism of action, and treatment using a novel nasal spray delivery system.

### Central MADRS assessment by phone

In this study, MADRS scores were remotely assessed by independent raters that could have potentially reduced the sensitivity for detecting subtle differences in clinical response. Although the MADRS rating by telephone interview has been validated in non-Japanese population [43], cultural differences including the way Japanese patients express their symptoms of depression may have influenced the sensitivity for efficacy in some MADRS items over the phone.

As observed in the present study, a substantial and well-characterized placebo response, which is often larger than the drug-placebo difference is a major challenge in AD drug development. The failure rate in AD trials is nearly 50% globally [44, 45], and failed trials in Japanese patients have been reported for several major ADs currently approved in Japan as well as globally [46–50].

All esketamine doses evaluated in this study appeared to be safe and were tolerated in Japanese patients. Safety was consistent with the findings from the global clinical development program [12, 23]. There was a transient dose-related BP elevation immediately after esketamine administration that spontaneously resolved shortly, in-line with literature evidence [51]. Transient dissociative symptoms occurred shortly after esketamine administration, as reported previously [52, 53]. Most TEAEs reported in this study across esketamine groups were mild or moderate and there were no apparent dose-related safety issues. Serious TEAEs were reported at low rates. Incidences of TEAEs leading to discontinuation were consistent with those observed in the global Phase 3 studies [12, 23].

## Conclusions

This study was unable to show the efficacy of esketamine as an add-on to oral ADs in adult Japanese patients with TRD. Esketamine appeared safe and was tolerated in Japanese patients, with safety profile consistent with the global data. Given that several factors described above may have influenced or impacted the ability to detect efficacy in this study, further studies mitigating the potential cofounders and implementing measures to minimize placebo response are warranted.

## Data Availability

The data sharing policy of Janssen Pharmaceutical Companies of Johnson & Johnson is available at https://www.janssen.com/clinical-trials/transparency. As noted on this site, requests for access to the study data can be submitted through Yale Open Data Access (YODA) Project site at http://yoda.yale.edu.

## Abbreviations

AD: Antidepressant
AE: Adverse event
ANCOVA: Analysis of covariance
BP: Blood pressure
BPRS+: Brief Psychiatric Rating scale
CADSS: Clinician Administered Dissociative States Scale
CGI-S: Clinical Global Impression-Severity Scale
CI: Confidence interval
C-SSRS: Columbia-Suicide Severity Rating Scale
DB: Double-blind
DSM-5: Diagnostic and Statistical Manual of Mental Disorders (fifth edition)
ECG: Electrocardiogram
E_max_: The maximum effect attributable to the drug
Esk: Esketamine
EU: European Union
FAS: Full analysis set
LOCF: Last observation carried forward
LS: Least-squares
MADRS: Montgomery-Asberg Depression Rating Scale
MCP-Mod: Multiple comparison procedure-modeling
MDD: Major depressive disorder
MedDRA: Medical Dictionary for Regulatory Activities
MGH-ATRQ: Massachusetts General Hospital-Antidepressant Treatment Response Questionnaire
MMRM: Mixed effect model using repeated measures
MOAA/S: Modified Observer’s Assessment of Alertness/Sedation
NMDA: N-methyl-D-aspartate
OL: Open-label
PWC-20: Physician Withdrawal Checklist 20-item
SD: Standard deviation
SDS: Sheehan Disability Scale
SE: Standard error
TEAE: Treatment-emergent adverse event
TRD: Treatment-resistant depression
US: United States
